# Independent clinic-based evaluation of dual POCTs for screening for HIV and syphilis in men who have sex with men in Italy, Malta, Peru, and the United Kingdom

**DOI:** 10.1186/s12879-024-09019-3

**Published:** 2024-02-29

**Authors:** Nigel Sherriff, Massimo Mirandola, Ronaldo Silva, Maddalena Cordioli, Alexandra Sawyer, Lorenzo Gios, Antonella Zorzi, Jorg Huber, Jaime Vera, Daniel Richardson, Mohammed Hassan-Ibrahim, Dominika Wlazly, Valeska Padovese, Christopher Barbara, Anabel Darmanin, Aaron Schembri, Carlos Caceres, Silver Vargas, Karel Blondeel, James Kiarie, Firdavs Kurbonov, Rosanna W. Peeling, Soe Soe Thwin, Igor Toskin, other Editorial Board Members of the ProSPeRo Network, Amina Hançali, Hicham Oumzi, Simon Mwima, Peter Kyambadde, Isaac Ssewanyana

**Affiliations:** 1 School of Sport and Health Sciences, University of Brighton, Village Way, Falmer, BN1 9PH UK; 2 Infectious Diseases Section, Department of Diagnostics and Public Health, University of Verona, Verona, Italy; 3 Microbiology and Virology Unit, Molecular Biology Department, Padua University Hospital, Padua, Italy; 4 Brighton & Sussex Medical School, University of Sussex, Brighton, UK; 5 Department of Microbiology & Infection, Royal Sussex County Hospital, University Hospitals Sussex NHS Foundation Trust, Brighton, UK; 6 Royal Sussex County Hospital Brighton, CIRU Research Laboratory, Brighton, UK; 7 Department of Dermatology and Venereology, Genito-Urinary Clinic, Mater Dei Hospital, Msida, Malta; 8 Pathology Department, Mater Dei Hospital, Msida, Malta; 9 Infectious Diseases Unit, Mater Dei Hospital, Msida, Malta; 10 Centro de Investigación Interdisciplinaria en Sexualidad, Sida y Sociedad, Universidad Peruana Cayetano Heredia, Lima, Peru; 11 World Health Organization, Department of Sexual and Reproductive Health and Research (includes the UNDP/UNFPA/UNICEF/WHO/World Bank Special Programme of Research, Development and Research Training in Human Reproduction [HRP]), Geneva, Switzerland; 12 Faculty of Medicine and Health Sciences, Ghent University, Ghent, Belgium; 13 Department of Clinical Research, London School of Hygiene and Tropical Medicine, London, UK; 14 Centre for Transforming Sexuality and Gender, University of Brighton, Edward Street, Brighton, BN2 0JG UK

**Keywords:** HIV, Syphilis, Public Health, Point-of-Care-Tests, Men who have Sex with Men, Clinic-based evaluation

## Abstract

**Introduction:**

Globally, the incidence of HIV and syphilis can be reduced by the use of validated point of care tests (POCTs). As part of the WHO PRoSPeRo Network, we aimed to evaluate the performance, acceptability, and operational characteristics of two dual HIV/syphilis POCTs (Bioline HIV/Syphilis Duo (Abbott) and DPP® HIV-Syphilis assay (Chembio) for the screening of HIV and syphilis amongst men who have sex with men (MSM).

**Method and analyses:**

A cross sectional study of 2,577 MSM in Italy, Malta, Peru, and the United Kingdom (UK) presenting to seven clinic sites, were enrolled. Finger prick blood was collected to perform POCTs and results compared with standard laboratory investigations on venepuncture blood. Acceptability and operational characteristics were assessed using questionnaires. Diagnostic meta-analysis was used to combine data from the evaluation sites.

**Results:**

Based on laboratory tests, 23.46% (*n* = 598/2549) of participants were confirmed HIV positive, and 35.88% of participants (*n* = 901/2511) were positive on treponemal reference testing. Of all participants showing evidence of antibodies to *Treponema pallidum*, 50.56% (*n* = 455/900) were Rapid Plasma Reagin (RPR) test reactive. Of HIV positive individuals, 60.62% (*n* = 354/584) had evidence of antibodies to *T. pallidum*, and of these 60.45% (*n* = 214/354) exhibited reactive RPR tests indicating probable (co)infection. For Bioline POCT, pooled sensitivities and specificities for HIV were 98.95% and 99.89% respectively, and for syphilis were 73.79% and 99.57%. For Chembio pooled sensitivities and specificities for HIV were 98.66% and 99.55%, and for syphilis were 78.60% and 99.48%. Both tests can detect greater than 90% of probable active syphilis cases, as defined by reactive RPR and treponemal test results. These dual POCTs were preferred by 74.77% (*n* = 1,926) of participants, due to their convenience, and the operational characteristics made them acceptable to health care providers (HCPs).

**Conclusions:**

Both the Bioline and the Chembio dual POCT for syphilis and HIV had acceptable performance, acceptability and operational characteristics amongst MSM in the PRoSPeRo network. These dual POCTs could serve as a strategic, more cost effective, patient and healthcare provider (HCP) friendly alternative to conventional testing; in clinical and other field settings, especially those in resource-limited settings.

## Background

Rates of sexually transmitted infections (STIs) including HIV and syphilis continue to be problematic globally with more than one million new cases diagnosed every day [1]. The global prevalance and incidence rates of syphilis increased between 2016 and 2020 [2]. Whilst HIV incidence declined by 31% between 2010 and 2020, rates remain substantially behind the global target of fewer than 500,000 new HIV infections per year globally by 2020 [1]. Prevalence and incidence rates of HIV and other STIs including syphilis remain highest amongst key populations such as men who have sex with men (MSM) [3, 4]. A recent meta-analysis reported that the global pooled prevalence of syphilis amongst MSM from 2000–2020 was 7.5% compared to the most recent estimate of syphilis of 0.5% amongst men in the general population in 2016 [5]. Notably over the last two years, there has been a significant increase in rates of syphilis amongst MSM in high-income settings [6].

Effective prevention and control strategies for HIV and syphilis rely on the availability of sensitive diagnostic testing for early detection and diagnosis, and for the guidance of treatment and prevention of onward transmission [7, 8]. Whilst laboratory-based serological tests provide the diagnostic ‘gold-standard’ for HIV and syphilis, some of these tests are technically demanding requiring skilled staff, invasive procedures (venepuncture), and require laboratory equipment that may not always be widely available in resource-limited settings. Consequently, the World Health Organization (WHO) has recommended the use of point-of-care tests (POCTs) to diagnose HIV infection which can be used outside of typical clinical laboratories by non-laboratory trained healthcare providers [9, 10]. The last decade has seen considerable efforts to develop such new diagnostic tools including POCTs for HIV and other STIs, and many are now commercially available [11]. Rapid HIV POCTs are routinely implemented for HIV screening in the public health sector. However serological testing for syphilis (treponemal and non-treponemal) remains mostly laboratory-based owing to the complex interpretation of results of serological testing at various stages of the disease [12].

Nevertheless, dual POCTs for detecting antibodies to HIV and syphilis have been developed for use with venous whole blood, serum/plasma, or finger-prick capillary whole blood. As results are available in 15–20 min, these tests allow same-day testing and potentially referral and/or treatment. In addition to improving the accessibility of syphilis testing and treatment by integrating the detection of syphilis into HIV programmes [8], the use of dual POCTs simplifies training by using one test instead of separate tests, reduces storage and transportation costs, and reduces waste disposal [13]. However, whilst demonstrating encouraging performance compared to gold-standard reference tests in laboratory-based studies, there is limited data on their performance in the field [14, 15]. Clinical studies in real-world settings are important because the performance of POCTs, including positive predictive values (PPV) and negative predictive values (NPV), can be influenced by epidemiological and environmental factors. Operational characteristics as well as human factors (e.g. the ability to follow properly the POCT procedures such as correctly taking whole blood finger prick specimens, correct timing of adding buffers and accurate reading and interpretation of the results), can also influence the performance of a POCT [16]. Finally, whilst the WHO provides recommendations and guidance on the use of dual HIV/syphilis testing in antenatal care settings, there is a need to develop recommendations on the integration of dual HIV/syphilis testing in other key populations, such as MSM [17].

Consequently, evaluation of the performance of these dual POCTs in clinic-based settings and their acceptability to patients and healthcare providers is a high priority for the development and global uptake of POCTs for STIs as set out by the WHO during three technical consultations [18, 19]. The primary objectives of this current clinic-based evaluation were to assess: i) the performance of two dual POCTs (SD Bioline HIV/Syphilis Duo—Abbott Diagnostics, United States and Chembio Dual Path Platform (DPP®) HIV–Syphilis Assay—Chembio, United States) for the screening of HIV and syphilis amongst MSM using finger prick capillary whole blood compared to reference laboratory-based serum tests for HIV and syphilis (HIV 1/2 EIA and the treponemal reference test), and; ii) the minimal operational characteristics and acceptability of these dual HIV-syphilis POCTs for health care providers (HCPs) and users respectively. A secondary objective of the study was to explore the performance and the potential utility of these dual HIV-syphilis POCTs in better identifying probable active syphilis using a combination of the treponemal and non-treponemal tests as the comparator.

## Methods

### Study design

This clinic-based evaluation was a multi-site, cross-sectional study of MSM presenting at sexual health clinics for HIV/STI screening. Detailed study procedures and testing methodologies were based on a WHO standardised core protocol and have been presented elsewhere [20]. The study approach was underpinned by the WHO guideline recommendations for diagnostic tests [21] and aligned and compliant with the Quality Assessment of Diagnostic Accuracy Studies (QUADAS-2) and, as far as possible, the Standards for Reporting Studies of Diagnostic Accuracy (STARD) [22, 23].

### Study setting, population, and sample

Participants were enrolled at seven study sites between May 2018 and October 2020 (Table 1). Test confirmation and reference testing was supported by appropriate reference laboratories.

**Table 1:** Enrolment sites and reference testing in Italy, Malta, Peru, and the UK

Country	Site ID	Enrollment site	Start/End	Reference tests	Enrolled/maxium sample size
Italy	4001	Infectious Diseases and Tropical Medicine Unit	08-May-18; 08-Feb-19	*HIV*: Enzyme-Linked Immunosorbent Assay (ELISA) 4th Gen, if positive Western Blot (WB) *Syphilis*: *Treponema pallidum* Passive Particle Agglutination (TPPA) with titration & Rapid Plasma Reagin (RPR) with titration	284 [of 225]
Italy	4002	Screening Center for Communicable Diseases	10-May-18; 06-Feb-19		208 [of 225]
Malta^a^	4005	GU clinic, Mater Dei Hospital	26-Jun-19; 27-Feb-20	*HIV*: ELISA 4th Gen, if positive WB *Syphilis*: *Treponema pallidum* Hemagglutination Assay (TPHA) with titration & RPR with titration^b^	517 [of 700]
Peru	4006	Cerits Alberto Barton	09-Apr-19; 14-Sep-20	*HIV*: ELISA 4th Gen, ELISA 3rd Gen, if discordant WB *Syphilis*: Chemiluminescence Immunoassay (CLIA) & RPR with titration^c^	382 [of 325]
Peru	4007	Tahuantinsuyo Bajo Clinic	09-Apr-19; 25-Sep 20		517 [of 650]
Peru	4034	San José STI clinic	06-Feb-20; 06-Oct-20		133 [of 325]
UK	4032	Brighton & Hove Sexual Health and Contraception Service	08-Aug-18; 02-May-19	*HIV*: ELISA 4th Gen, ELISA 3rd Gen, if discordant WB *Syphilis*: TPPA with titration & RPR with titration^d^	538 [of 680]
**Totals**					**2577**

^a^Independent study, data-sharing agreement signed

^b^All have TPHA, part of those have been retested with TPPA in Italy

^c^CLIA on all samples was done in Italy

^d^Confirmation of HIV positives, TPPA and RPR on all samples was done in Italy

*MSM* aged ≥ 18 years attending one of the sexual health clinics at the study sites and who provided written informed consent were included in the study. HCPs who administered the POCTs were also included in the study (to complete a provider questionnaire). To be eligible, HCPs had to have been trained in, and administered, the POCTs under evaluation and provide written informed consent. Participants could only be enrolled in the study once. As set out in the core protocol [20], sample size calculations were based on the WHO/TDR (Special Programme for Research and Training in Tropical Diseases) expert panel document on the evaluation of new diagnostic methods and techniques [24] which takes into account the estimated performance of the POCTs and the seroprevalence of HIV/syphilis in MSM presenting to the clinics.

### POCTs under evaluation and reference laboratory tests

The POCTs evaluated were SD Bioline HIV/Syphilis Duo (Abbott Diagnostics, United States; hereafter termed SD Bioline) and Chembio Dual Path Platform (DPP®) HIV–Syphilis Assay (Chembio, United States; hereafter termed Chembio). Both are single-use qualitative immunochromatographic assays for the simultaneous detection of antibodies including anti-HIV and anti-*Treponema pallidum* (syphilis) in human serum, plasma, whole venous or fingerpicked blood. The Chembio company developed the DPP Micro Reader (MR) to minimise error due to subjective visual interpretation which can be fitted to the POCT via a dedicated holder. It scans the cartridge and verifies the presence of line(s) at the control and each of the test line positions. Results from both POCTs (including the results from the MR) were compared with those of the HIV and syphilis serological laboratory standard assays or ‘gold-standard’ tests. Respectively these were laboratory-based HIV 1/2 EIA confirmed by immunoblot (antibody only) or equivalent, while the reference test for syphilis was the *Treponema pallidum* Passive Particle Agglutination (TPPA) or equivalent. In Malta the *Treponama pallidum* hemagglutination assay TPHA was used instead of TPPA, which can be slightly less sensitive. In Peru, the reference testing was done with TPHA, but was later retested with a chemiluminescence assay (CLIA) for *Treponema pallidum* specific antibodies detection in Italy due to technical challenges with TPHA testing in Peru. It has been shown that the performance of TPPA and CLIA are equivalent [25]. Reference tests were performed in accordance with the manufacturer’s directions and laboratory staff were blinded to the POCT results. External quality assessment (EQA) for the HIV and syphilis testing at both the reference laboratories (proficiency panels) and associated POCT sites (Dried Tube Specimens [DTS]) was supported by the USA Centers for Disease Control and Prevention (CDC) to monitor the quality of both reference and clinic based POCT testing [26].

### Procedural steps (Fig. 1)

**Fig. 1:**
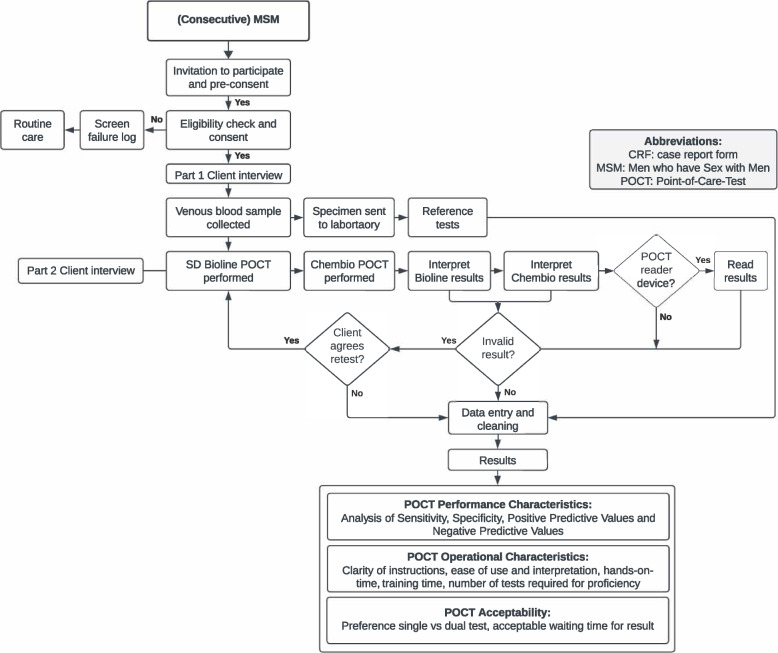
Recruitment and data collection flowchart

Eligible participants were enrolled consecutively following written informed consent. Each participant was assigned a unique study identification number, which was delinked from all personal identifiers. A standardised paper-based structured questionnaire was then used by the HCP to collect information on patient demographics, clinical characteristics (medical history), and acceptability regarding the index POCTs (e.g. preferences for stand-alone or dual HIV/ syphilis testing). Two sample types were then collected: 1) blood collection by venepuncture for serum collection and 2) capillary blood collection by finger-prick method. For venous blood, 3–5 ml was collected by trained clinical staff, and transported for reference testing in accordance with standard operating procedures at each local site. For finger prick samples, the manufacturers’ instructions were followed step-by-step, collecting the required amount of capillary blood using the collection devices provided in both test kits and reading the test result within the reading window (measured with a timer for each test). A double reader method (Reader 1-Reader 2 [R1-R2]) was adopted for the POCTs to determine variability in the interpretation of test results. Readers were independent and blind to each other (including to any previous syphilis history) and were either doctors or nurses in the clinical setting who had been trained specifically in specimen collection and handling including performance and reading of the POCTs as well as familiarisation with the study standard operating procedures. A separate provider questionnaire was completed at the end of the study period by HCPs who performed the two POCTs. These provided information on the POCTs operational characteristics.

### Acceptability and operational characteristics

Acceptability data was obtained from participant responses by asking: if these tests were available in the future, how long would they be willing to wait for their results at the clinic, and whether they preferred two single tests for HIV and syphilis detection or one dual test. Regarding operational characteristics, the kits were rated by clinic staff (HCPs) who performed the POCTs using an eight-item provider completed questionnaire (clarity of kit instructions, ease of POCT use, ease of interpretation, rapidity of testing, hands on time, training time required, number of tests performed to achieve competency/proficiency, and overall comment/recommendation [free text response]).

### Statistical analysis

Diagnostic meta-analysis was used to combine data from the seven evaluation sites using a random effects model. Pooled and site values for sensitivity, specificity, PPV, and NPV as well as positive and negative likelihood ratios (LR + and LR-) for each rapid test were estimated. POCT results were compared with the laboratory test results (see Table 1); namely for HIV POCT result versus laboratory-based HIV EIA and/or immunoblot or equivalent, and for syphilis POCT treponemal versus laboratory-based treponemal (TPPA or equivalent) for the primary objective, and a combination of the latter and non-treponemal (RPR) for the secondary objective. Concordance between R1-R2 readings was estimated by calculating percentage agreement (concordance) and Cohen’s κ (κ for binary variables) [27, 28]. Forest plots of the performance characteristics of the POCTs (sensitivity, specificity) for HIV and syphilis are depicted.

For operational characteristics of the assays including acceptability, the proportion of patients preferring dual vs single testing was analysed as well as the acceptable waiting time for results. For HCPs per POCT, the proportion of responses to each category regarding clarity of kit instructions, ease of use and ease of interpretation were noted, together with analyses relating to hands-on time required, rapidity of test result, training time required, and the number of tests required to achieve proficiency. STATA V.16.1 (College Station, TX: StataCorp LP) was used for data management, and SAS V. 9.4 (SAS Institute Inc., Cary, NC, USA) for all statistical analyses.

## Results

### Study population

A total sample of 2,577 MSM were enrolled in the study with an average travelling time from home to the clinical site of 40 min. The mean age of the participants was 36.19 years (median: 34; SD 12; min. 18, max. 79). In terms of the overall sample, 84.27% (*n* = 2,170) had previously been tested for syphilis and 34.82% (*n* = 751) reported a previous syphilis diagnosis before study enrolment (Table 2). 94.18% (*n* = 2,426) reported previously being tested for HIV, and 21.37% (*n* = 518) reported being positive for HIV. Participants provided bio-behavioural information via a structured interview, fingerstick whole blood for HIV/syphilis rapid testing, and venous whole blood for HIV and syphilis serological testing.

**Table 2:** Clinical characteristics of participants

Variable	Category	Pooled		Italy		Malta		Peru		UK	
		**N**	**%**	**N**	**%**	**N**	**%**	**N**	**%**	**N**	**%**
***SYPHILIS***											
**Previously tested for syphilis**	**No**	376	14.60	118	23.98	61	11.84	179	17.34	18	3.36
	**Yes**	2170	84.27	369	75.00	450	87.38	841	81.49	510	95.15
	**N/A**	9	0.35	0	0	0	0	7	0.68	2	0.37
	**Don't know**	17	0.66	5	1.02	4	0.78	5	0.48	3	0.56
	**Don’t want to answer**	3	0.12	0	0	0	0	0	0	3	0.56
	**Missing**	2	0.08	0	0	0	0	0	0	2	0.37
**Previously diagnosed for syphilis**	**No**	1406	65.18	211	57.18	339	75.50	495	59.07	361	72.06
	**Yes**	751	34.82	158	42.82	110	24.50	343	40.93	140	27.94
	**Missing**	420	16.30	123	0.25	66	12.82	194	18.80	37	6.88
***HIV***											
**Previously tested for HIV**	**No**	142	5.51	32	6.50	34	6.60	68	6.59	8	1.49
	**Yes**	2426	94.18	459	93.29	481	93.40	953	93.31	523	97.39
	**N/A**	2	0.08	0	0	0	0	1	0.10	1	0.19
	**Don’t know**	2	0.12	1	0.20	0	0	0	0	2	0.37
	**Don’t want to answer**	3	0.12	0	0	0	0	0	0	3	0.56
	**Missing**	1	0.04	0	0	0	0	0	0	1	0.19
**Last HIV test**	** < 1 year**	1564	64.57	285	62.09	326	67.92	617	64.07	336	64.62
	** ≥ 1 year**	858	35.43	174	37.91	154	32.08	346	35.93	184	35.38
	**Missing**	155	6.01	33	6.71	35	6.80	69	6.69	18	3.35
**Result of last HIV test**	**Negative**	1900	78.38	411	89.54	423	88.13	689	71.62	377	72.08
	**Positive**	518	21.37	47	10.24	57	11.88	269	27.96	145	27.72
	**Indeterminate**	3	0.12	0	0	0	0	3	0.31	0	0
	**Don’t know**	2	0.08	1	0.22	0	0	0	0	1	0.19
	**Don’t want to answer**	1	0.04	0	0	0	0	1	0.10	0	0
	**Missing**	153	5.94	33	6.71	35	6.80	70	6.78	15	2.79

### Results of the laboratory-based (reference) testing

Based on the reference test algorithm, 23.46% (*n* = 598/2549) of all participants were confirmed HIV positive, and 35.88% of all participants (*n* = 901/2511) were positive on treponemal reference testing. Of all participants showing evidence of antibodies to *T. pallidum*, 50.56% (*n* = 455/900) were found to be reactive on RPR testing. Of those who were HIV positive, 60.62% (*n* = 354/585) also had evidence of antibodies to *T. pallidum*. Amongst the latter, 60.45% (*n* = 214/354) exhibited reactive RPR tests [29].

### Performance of the POCTs

#### HIV testing

The pooled sensitivity and specificity of the HIV component of the Bioline testing kit were 98.95% (95% CI = 96.83–99.66) and 99.89% (95% CI = 98.48–99.99) respectively (Table 3; see also Table 4). PPVs for minimum and maximum HIV prevalence scenarios from sites was 99.07% at 10.6% minimum prevalence and 99.80% at 35.6% maximum prevalence. NPVs at the minimum prevalence scenario were 99.88% and 99.42% at the maximum prevalence. The LR + for Bioline was 925.95 (64.03–13390.24) and the LR- was 0.01 (0.00–0.03). For Chembio, the pooled sensitivity and specificity were 98.66% (95% CI = 96.15–99.54) and 99.55% (95% CI = 98.79–99.84) respectively. The PPV at the 10.60% minimum prevalence was 96.30% and at the 35.60% maximum prevalence was 99.18%. NPV were 99.84% and 99.26% respectively. The LR + for Chembio was 220.75 (81.47–598.15), and the LR- was 0.01 (0.01–0.04). Using the micro reader (MR), the pooled sensitivity and specificity for Chembio were 98.09% (95% CI = 95.35–99.23) and 99.54% (95% CI = 99.11–99.76) respectively. PPV for the minimum and maximum prevalence scenarios were 96.20% at the 10.60% minimum prevalence and 99.16% at 35.60% maximum prevalence. NPV at the minimum prevalence scenario was 99.77% and 98.95% at the maximum prevalence. Using the MR, the LR + 211.61 (103.69–431.89), and the LR- was 0.02 (0.01–0.04). The agreement of testing results as read by two readers was high and statistically significant for both Bioline and Chembio with kappa statistics of 0.98 (95% CI = 0.96–0.99) and 0.98 (95% CI = 0.98–0.99) respectively.

**Table 3:** Pooled performance characteristics of POCTs for HIV compared to reference assays

POCT	Pooled Sensitivity	Pooled Specificity	Prevalence Scenarios^a^	PPV	NPV
**Bioline**	98.95%	99.89%	5.60%	98.16%	99.94%
			10.60%	99.07%	99.88%
			35.60%	99.80%	99.42%
			40.60%	99.84%	99.29%
**Chembio**	98.66%	99.55%	5.60%	92.86%	99.92%
			10.60%	96.30%	99.84%
			35.60%	99.18%	99.26%
			40.60%	99.34%	99.09%
**Chembio MR (Micro Reader)**	98.09%	99.54%	5.60%	92.67%	99.89%
			10.60%	96.20%	99.77%
			35.60%	99.16%	98.95%
			40.60%	99.32%	98.70%

^a^Actual prevalence range (min–max from sites): 10.6%-35.6%

**Table 4:** Performance characteristics of POCTs for HIV compared to reference assays by site

	**Bioline**							**Chembio**							**Chembio (MR)**						
**Sites**	**I**	**P**	**N**	**FP**	**FN**	**Sensitivity (%)**	**Specificity (%)**	**I**	**P**	**N**	**FP**	**FN**	**Sensitivity (%)**	**Specificity (%)**	**I**	**P**	**N**	**FP**	**FN**	**Sensitivity (%)**	**Specificity (%)**
4001 – Italy 1	0	31	252	1	0	100 (88.43–100)	99.60 (97.82–99.99)	0	35	248	5	0	100 (88.43–100)	98.02 (95.45–99.36)	0	32	251	2	0	100 (88.43–100)	99.21 (97.17–99.90)
4002 – Italy 2	0	24	184	0	0	100 (85.75–100)	100 (98.02–100)	1	24	183	0	0	100 (85.75–100)	100 (98.00–100)	1	24	183	1	1	95.83 (78.88–99.89)	99.45 (96.99–99.99)
4005 – Malta	0	66	448	0	3	95.65 (87.82–99.09)	100 (99.17–100)	0	64	450	0	5	92.75 (83.89–97.61)	100 (99.17–100)	0	64	450	1	6	91.30 (82.03–96.74)	99.78 (98.75–99.99)
4006 – Peru 1	0	119	257	0	1	99.17 (95.44–99.98)	100 (98.57–100)	0	120	256	1	1	99.17 (95.44–99.98)	99.61 (97.84–99.99)	4	118	254	1	1	99.15 (95.37–99.98)	99.61 (97.83–99.99)
4007 – Peru 2	0	168	345	0	1	99.41 (96.75–99.99)	100 (98.93–100)	0	169	344	1	1	99.41 (96.75–99.99)	99.71 (98.39–99.99)	0	168	345	0	1	99.41 (96.75–99.99)	100 (98.93–100)
4032 – UK	1	138	382	1	0	100 (97.34–100)	99.74 (98.55–99.99)	0	140	381	3	1	99.28 (96.03–99.98)	99.22 (97.73–99.84)	0	139	383	2	2	98.56 (94.90–99.83)	99.48 (98.13–99.94)
4034 – Peru 3	0	54	78	9	2	95.74 (85.46–99.48)	89.41 (80.85–95.04)	0	46	86	1	2	95.74 (85.46–99.48)	98.82 (93.62–99.97)	0	47	85	2	2	95.74 (85.46–99.48)	97.65 (91.76–99.71)
Fixed effect model	1	600	1946	11	7	98.83 (97.60–99.53)	99.44 (98.99–99.72)	1	598	1948	11	10	98.33 (96.73–99.15)	99.44 (98.93–99.70)	5	592	1951	9	13	97.82 (96.09–98.79)	99.54 (99.06–99.77)
**Meta-analysis (random effects model)**						**98.95 (96.83–99.66)**	**99.89 (98.48–99.99)**						**98.66 (96.15–99.54)**	**99.55 (98.79–99.84)**						**98.09 (95.35–99.23)**	**99.54 (99.11–99.76)**
	Between sites variability—SD (*p*-value)					8.04 (0.5223)	9.99 (0.2529)	Between sites variability—SD (*p*-value)					8.30 (0.3436	8.08 (0.4099	Between sites variability—SD (*p*-value)					8.13 (0.3031)	[fixed effect for specificity)
					DOR	87,728.21 (3897.99–1,974,412.05)						DOR	16,415.20 (3757.28–71,716.48)							DOR	9656.87 (3789.06–24611.69)
					LR +	925.95 (64.03–13390.24)						LR +	220.75 (81.47–598.15)							LR +	211.61 (103.69–431.89)
					LR-	0.01 (0.00–0.03)						LR-	0.01 (0.01–0.04)							LR-	0.02 (0.012–0.04)

*I* Invalid, *P* Positive, *N* Negative, *FP* False Positive, *FN* False Negative, *DOR* Diagnostic Odds Ratio, *LR* Likelihood Ratio

**Fig. 2:**
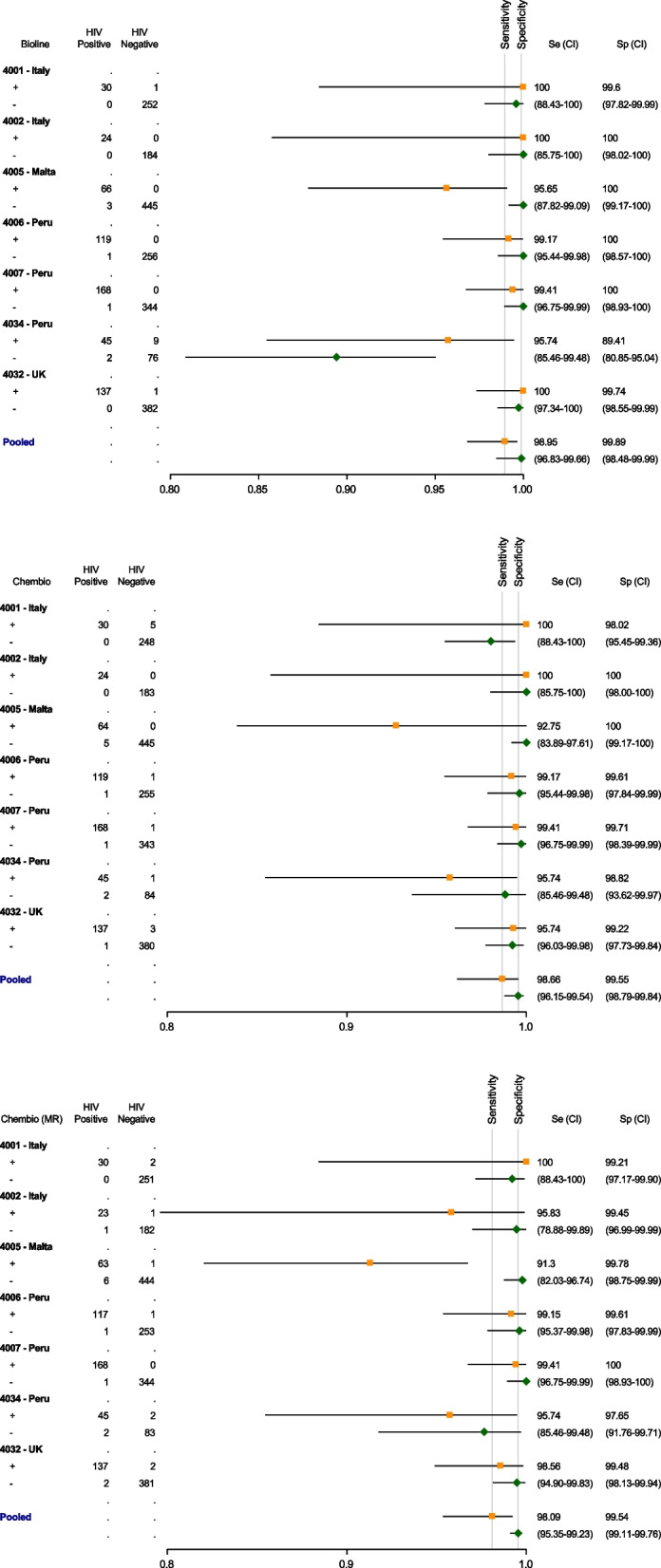
Performance characteristics of Bioline and Chembio (including the micro reader) for HIV compared to reference assays (per site)

The sensitivities and specificities by site of the HIV component of the Bioline and Chembio testing kits (including MR) are shown in Table 4, and in Fig. 2 via forest plots. The orange squares represent the point estimate for sensitivity and the green diamonds represent the point estimate for specificity from each study site. The horizontal lines represent the 95% confidence intervals.

As shown in Table 4 and in Fig. 2, the specificity of the HIV component for the Bioline test in the 4034 Peru site was lower than other sites with an unexpectedly high number of false positives (*n* = 9) compared to the reference test.

### Syphilis testing

Pooled sensitivity and specificity for the syphilis component of the Bioline dual test kit were 73.79% (95% CI = 63.98–81.70) and 99.57% (95% CI = 99.09–99.79) respectively (Table 5; see also Table 6). PPV for minimum and maximum syphilis prevalence scenarios from sites was 98.18% at 23.88% minimum prevalence and 99.50% at 53.79% maximum prevalence. NPV at the minimum prevalence scenario was 92.37% and 76.55% at the maximum prevalence. The LR + for Bioline was 169.72 (80.18–359.25), and the LR- was 0.26 (0.19–0.37). For Chembio, the pooled sensitivity and specificity were 78.60% (95% CI = 69.73–85.41) and 99.48% (95% CI = 98.69–99.80). PPV at the 23.88% minimum prevalence was 97.93% and at 53.79% maximum prevalence was 99.43% while the NPVs were 93.68% and 76.52% respectively. The LR + for Chembio was 80.72 (42.48–153.37), and the LR- was 0.20 (0.14–0.28). Using the Chembio MR, the pooled sensitivity and specificity for Chembio were 81.10% (95% CI = 72.30–86.13) and 99.01% (95% CI = 98.13–99.48). PPV for the minimum and maximum prevalence scenarios was 96.21% at the 23.88% minimum prevalence and 98.95% at 53.79% maximum prevalence. NPV at the minimum prevalence scenario was 94.07% and 81.04% at the maximum prevalence. Using the MR, the LR + 151.47 (57.41–399.65), and the LR- was 0.215 (0.15–0.31). For syphilis, the agreement of testing results as read by two readers was (as with the HIV component) also high and statistically significant for Bioline and Chembio with kappa statistics of 0.97 (95% CI = 0.97–0.99) and 0.98 (95% CI = 0.97–0.99) respectively.

**Table 5:** Pooled performance characteristics of POCTs for syphilis compared to reference assays

**POCT**	**Pooled Sensitivity**	**Pooled Specificity**	**Prevalence Scenarios** ^**a**^	**PPV**	**NPV**
**Bioline**	73.79%	99.57%	18.88%	97.56%	94.23%
			23.88%	98.18%	92.37%
			53,79%	99.50%	76.55%
			58,79%	99.59%	72.70%
**Chembio**	78.60%	99.48%	18.88%	97.24%	95.23%
			23.88%	97.93%	93.68%
			53,79%	99.43%	79.97%
			58,79%	99.54%	76.52%
**Chembio MR (Micro Reader)**	80.10%	99.01%	18.88%	94.96%	95.53%
			23.88%	96.21%	94.07%
			53,79%	98.95%	81.04%
			58,79%	99.14%	77.72%

^a^Actual prevalence range (min–max from sites): 23.88%-53,79%

**Table 6:** Performance characteristics of POCTs for syphilis compared to reference assays by site

**Sites**	**Bioline**							**Chembio**							**Chembio (MR)**						
	**I**	**P**	**N**	**FP**	**FN**	**Sensitivity (%)**	**Specificity (%)**	**I**	**P**	**N**	**FP**	**FN**	**Sensitivity (%)**	**Specificity (%)**	**I**	**P**	**N**	**FP**	**FN**	**Sensitivity (%)**	**Specificity (%)**
4001 – Italy 1	0	74	210	0	20	78.72 (69.07–86.49)	100 (98.08–100)	0	77	207	0	17	81.91 (72.63–89.10)	100 (98.08–100)	0	74	210	0	20	78.72 (69.07–86.49)	100 (98.08–100)
4002 – Italy 2	0	56	152	1	21	72.37 (60.91–82.01)	99.24 (95.85–99.98)	1	61	146	1	16	78.95 (68.08–87.46)	99.24 (95.82–99.98)	1	60	147	1	17	77.63 (66.62–86.40)	99.24 (95.82–99.98)
4005 – Malta	0	81	434	3	45	63.41 (54.25–71.91)	99.23 (97.78–99.84)	0	88	427	6	41	66.67 (57.60–74.91)	98.47 (96.70–99.44)	0	97	418	7	33	73.17 (64.43–80.76)	98.21 (96.36–99.28)
4006 – Peru 1	0	163	210	0	24	87.17 (81.51–91.60)	100 (98.04–100)	0	167	206	0	20	89.30 (83.97–93.34)	100 (98.04–100)	4	167	202	1	18	90.22 (84.98–94.10)	99.46 (97.03–99.99)
4007 – Peru 2	0	179	335	1	48	78.76 (72.85–83.91)	99.65 (98.08–99.99)	0	194	320	1	33	85.40 (80.11–89.73)	99.65 (98.08,99.99)	0	196	318	2	32	85.84 (80.60–90.11)	99.31 (97.51–99.92)
4032 – UK	0	60	425	1	65	47.58 (38.54–56.74)	99.72 (98.47–99.99)	0	71	414	2	55	55.65 (46.45–64.56)	99.45 (98.01–99.93)	0	77	408	3	50	59.68 (50.49–68.39)	99.17 (97.59–99.83)
4034 – Peru 3	0	56	75	1	15	78.57 (67.13–87.48)	98.36 (91.20–99.96)	0	58	73	1	13	81.43 (70.34–89.72)	98.36 (91.20–99.96)	0	63	68	3	10	85.71 (75.29–92.93)	95.08 (86.29–98.97)
Fixed effect model	0	669	1841	7	238	73.56 (70.28–76.59)	99.57 (99.03–99.81)	1	716	1793	11	195	78.33 (75.24–81.13)	99.32 (98.70–99.64)	5	734	1771	17	180	79.93 (76.91–82.65)	98.94 (98.23–99.37)
**Meta-analysis (random effects model)**						**73.79 (63.98–81.70)**	**99.57 (99.09–99.79)**						**78.60 (69.73–85.41)**	**99.48 (98.69–99.80)**						**80.10 (72.30–86.13)**	**99.01 (98.13–99.48)**
	Between sites variability—SD (*p*-value)					7.64 (0.0946)	_	Between sites variability—SD (*p*-value)					7.64 (0.0967)	7.54 (0.5719)	Between sites variability—SD (*p*-value)					7.55 (0.1097)	7.34 (0.7180)
				DOR		244.05 (268.89–1546.09)						DOR	401.59 (185.03–871.63)						DOR	704.14 (216.28–2292.50)	
				LR +		169.72 (80.18–359.25)						LR +	80.72 (42.48–153.37)						LR +	151.47 (57.41–399.65)	
				LR-		0.26 (0.19–0.37)						LR-	0.20 (0.14–0.28)						LR-	0.22 (0.15–0.31)	

*I* invalid, *P* Positive, *N* Negative, *FP* False Positive, *FN* False Negative, *DOR* Diagnostic Odds Ratio, *LR* Likelihood Ratio

**Fig. 3:**
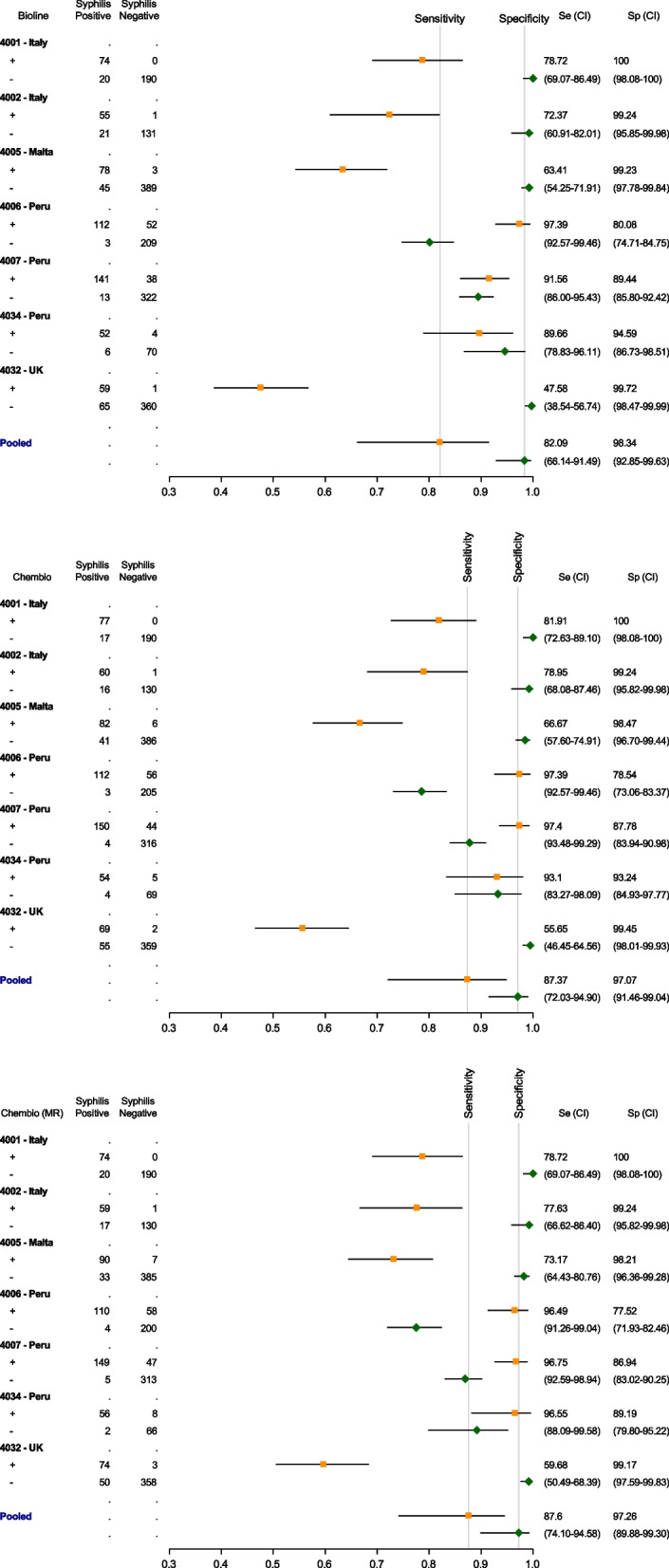
Performance characteristics of Bioline and Chembio (including the micro reader) for syphilis compared to reference assays (per site)

The sensitivities and specificities by site of the syphilis component of the two index test kits can be seen in Table 6, and in Fig. 3 via forest plots.

As shown in Table 6 and in Fig. 3, the sensitivities of the treponemal component for both the Bioline and Chembio tests were low in the UK. The Bioline sensitivity was 47.58% (95% CI = 38.54–56.74) although specificity was high 99.72% (95% CI = 98.47–99.99). Similarly, for Chembio, the sensitivity for the UK site was 55.65% (95% CI = 46.45–64.56) although specificity was again high 99.45% (95% CI = 98.01–99.93). The Chembio MR sensitivity was 59.68% (95% CI = 50.49–68.39), and specificity of 99.17% (95% CI = 97.59–99.83).

### TPPA, RPR, and titration values

A secondary objective of the study was to explore the performance and the potential utility of the POCTs to better identify active syphilis infection using a combination of the treponemal and non-treponemal tests as the comparator. In Table 7, positive treponemal reference test (TPPA/TPHA) results are presented and compared with their respective POCT results. For reasons of presentation and space, in the first column TPPA titres are collapsed into three categories for RPR positives and three categories for RPR negatives. The percentage agreement against the TPPA reference test is then provided for each POCTs. As can be seen, when TPPA and RPR are both positive with high titres, the POCTs perform well against the reference test. When the TPPA is positive and RPR is negative, the higher the TPPA titre, the better the performance of the POCTs’ treponemal component. Peruvian data are not included in this analysis.

**Table 7:** Syphilis POCTs results compared to TPPA and RPR titres (does not include Peruvian sites)

Titres			POCT result % agreement with reference test		
**TPPA + **	**RPR**		**Bioline**	**Chembio**	**Chembio MR**
** ≥ 20,480**	** + **	256			
		128	100% (1/1)	100% (1/1)	100% (1/1)
		64	100% (3/3)	100% (3/3)	100% (3/3)
		32	100% (3/3)	100% (3/3)	100% (3/3)
		16	100% (5/5)	100% (5/5)	100% (5/5)
		8	100% (5/5)	100% (5/5)	100% (5/5)
		4	100% (9/9)	100% (9/9)	88.89% (8/9)
		2	92.86% (13/14)	92.86% (13/14)	85.71% (12/14)
		1	100% (3/3)	100% (3/3)	100% (3/3)
**2560–10240**	** + **	256	100% (44)	100% (4/4)	100% (4/4)
		128			
		64	100% (4/4)	100% (4/4)	100% (4/4)
		32	100% (6/6)	100% (6/6)	100% (6/6)
		16	100% (5/5)	100% (5/5)	100% (5/5)
		8	90% (9/10)	90% (9/10)	95.24% (10/10)
		4	100% (13/13)	100% (13/13)	100% (13/13)
		2	90.91% (10/11)	100% (11/11)	100% (11/11)
		1	94.74% (18/19)	94.74% (18/19)	89.47% (17/19)
**80–1280**	** + **	256			
		128			
		64	100% (1/1)	100% (1/1)	100% (1/1)
		32	100% (1/1)	100% (1/1)	100% (1/1)
		16			
		8	100% (1/1)	100% (1/1)	100% (1/1)
		4	75% (3/4)	100% (4/4)	100% (4/4)
		2	50% (6/12)	50% (6/12)	66.67% (8/12)
		1	45.45% (5/11)	45.45% (5/11)	54.55% (6/11)
** ≥ 20,480**	**-**	0	87.10% (27/31)	87.10% (27/31)	87.10% (27/31)
**2560–10240**	**-**	0	73.74% (73/99)	79.80% (79/99)	82.83% (82/99)
**80–1280**	**-**	0	27.66% (39/141)	36.17% (51/141)	40.43% (57/141)

### Operational characteristics

Most participants preferred one dual test for HIV and syphilis infection rather than two single tests (74.77%; *n* = 1,926) and almost all were willing to wait for the results if the dual POCT tests were available at their clinic in the future (95.61%; *n* = 2,462). Of those willing to wait, 92.24% (*n* = 2,270) indicated that they would be willing to wait for at least 20 min and up to two hours for their results. As the test results are available within 15 min, this provides an important opportunity for referral to treatment.

**Fig. 4:**
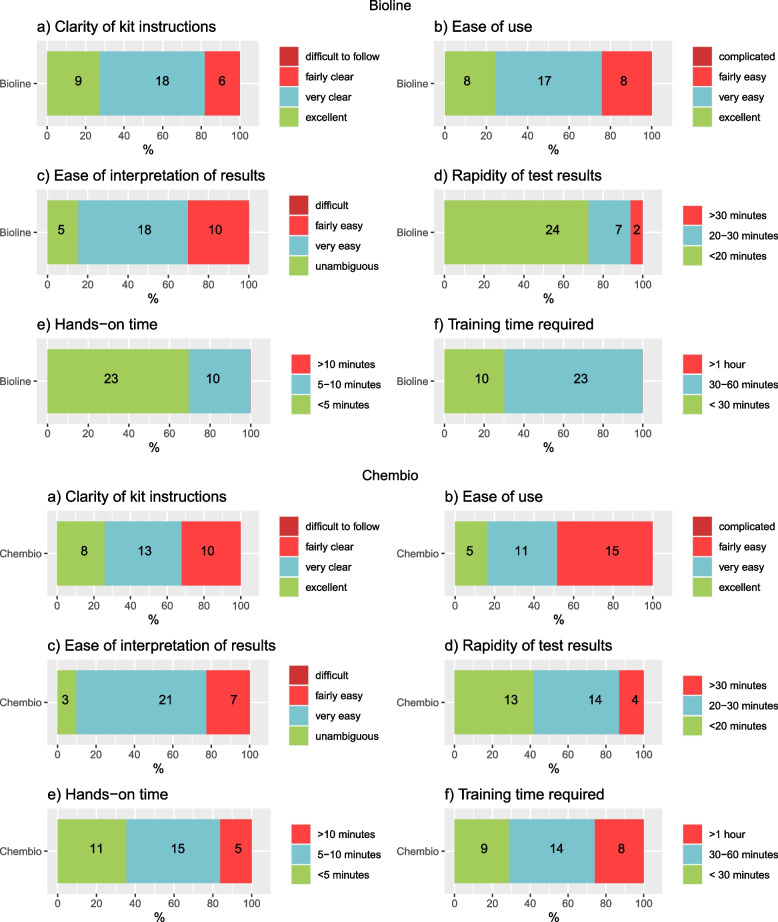
Operational characteristics of two dual rapid diagnostic tests for HIV/syphilis

Thirty-three clinic staff self-completed the survey on the dual tests’ operational characteristics. Clarity of kit instructions and ease of use were reported to be ‘very clear’, ‘very easy’ or ‘excellent’ for over 70% of providers for Bioline POCT and slightly lower (between 50%-60%) for Chembio POCT. Ease of interpretation of results was reported by 100% of providers as being ‘fairly easy’, ‘very easy’ or’unambiguous’ for both Bioline and Chembio POCTs. Most providers reported that test results were available in 30 min or less (94% and 83%), with hands-on-time of 5 min or less (100% and 84% for Bioline and Chembio respectively). For training time required, all providers reported needing less than one hour for Bioline training and approximately one in four providers reported needing more than one hour for Chembio training (Fig. 4).

## Discussion

The purpose of the study was to assess the performance characteristics, acceptability to end-users, and operational characteristics for HCPs of two dual HIV/syphilis POCTs for the screening for HIV and syphilis amongst MSM presenting at sexual health clinics for HIV/STI screening in Italy, Malta, Peru, and the UK. Overall, when compared to reference testing for HIV detection, both Bioline and Chembio dual testing kits performed well with similar performance in terms of high sensitivities and specificities across all seven study sites. Pooled sensitivity for Bioline was 98.95%, 98.66% for Chembio, and 97.82% for the Chembio MR. Pooled specificity was 99.89% (Bioline), 99.55% (Chembio), and 99.54% (Chembio MR). Pooled results also indicated good performance in both high and low HIV prevalence scenarios. Such findings overall are broadly in line with other studies using the same test kits, similar study settings, population (MSM) and sample type (whole blood rather than serum) [30–32]. As the WHO recommends a sensitivity and specificity of 99% and 98% respectively for HIV POCTs [33] in our clinic-based evaluation both Bioline and Chembio are acceptable with regards to fulfilling these criteria, and would thus potentially be a suitable option to use clinically and in screening programmes in the diagnosis of HIV infection which may be particularly beneficial in resource-limited settings [34].

Of note is that the specificity of the HIV component for the Bioline tests in the 4034 Peru site was lower than other sites with an unexpectedly high number of false positives (*n* = 9) compared to the reference test. Three of these false positive cases were not confirmed by the independent second reader, suggesting a possible faint HIV line or an error in result reporting. On further investigation, the problem does not seem to be caused by one specific provider, as the first reader for those cases was not always the same. There may have been a technical error, but the other sites in Peru (4006, 4007) used the same batch of tests without encountering such problems. A potential explanation is that these participants may have also been recruited in a HIV-vaccine trial that was running within the same population in Lima at the same time. However, it cannot be confirmed whether the false positive cases in this study also participated in the HIV-vaccine trial study.

When compared to reference testing for anti-treponemal antibody detection, sensitivity for both index tests were (as expected) lower than for the HIV component although remaining satisfactory with high specificity. This lower sensitivity was expected since infection with *T. pallidum* usually elicits a significantly lower antibody response when compared to that of HIV and therefore fainter test line signals. The pooled sensitivities recorded were 73.79% for Bioline, 78.6% for Chembio, and 80.1% for the Chembio MR. Pooled specificities were 99.57% (Bioline), 99.48% (Chembio), and 99.01% (Chembio MR). These results are broadly in line with other studies using the same test kits, similar study settings, population and sample type [30, 32]. However, the sensitivity of the syphilis component in both the index tests in the UK (site 4032) was particularly low at 47.58% for Bioline, 55.65% for Chembio, and 59.68% for the Chembio MR. This means that for Bioline, over one half of the UK cases with proven prior exposure to syphilis (by exhibiting a positive TPPA reference test) remained undetected by this POCT. The Chembio test, whilst performing marginally better, still failed to detect just under one half of all UK proven positives.

Since the performance of both POCTs was more satisfactory at all other sites included in this multicentre study, the anomalous results obtained in the UK are surprising and clearly warrant further investigation. Systematic error in sample processing can be ruled out given all the reference laboratories and POCT testing sites were subject to EQA and Quality Control (QC). Results showed that the laboratories demonstrated high EQA performance. Both HIV/syphilis POCTs gave expected EQA results in the evaluation sites using dried tube specimens [26] suggesting the operating procedures by HCPs was also not a factor. Alternatively, it is possible to hypothesise that the MSM sample in the UK site may have been different to the samples from other sites. Whilst MSM more broadly are considered to be at greater risk of STIs compared to the general population, research indicates that HIV pre-exposure prophylaxis (HIV PrEP) use also increases this risk [35, 36]. During the study period, PrEP was readily available in the UK clinic site as a consequence of a national research trial of PrEP [37]. It is reasonable to assume that HIV PrEP users at the Brighton site may have exposed themselves more frequently to the risk of acquiring STIs including syphilis. Given the quarterly comprehensive STI screening that is required as a result of PrEP uptake, any individuals are treated almost immediately potentially compromising the magnitude of the antibody response. Moreover, a UK study highlighted the purchase and use of self-prescribing antibiotics by some MSM as pre-exposure or post-exposure prophylaxis for STI prevention (STI prophylaxis) [38]. To test for this possibility, we conducted a sensitivity analysis to explore antibiotic use in the previous three weeks prior to being tested and POCT performance. However, no clear pattern emerged suggesting antibiotic ‘interference’ is not an issue and can most likely be discounted as an explanation. We also examined whether due to the low HIV incidence as a result of the PrEP trial, the inclusion of known HIV positive people in the study (allowed by the core protocol and foreseen in the questionnaire), may have influenced the index tests’ performance through potential biological interference between HIV infected individuals and the syphilis component [39]. Again, sensitivity analysis indicated this is an unlikely explanation as sensitivity and specificity improved slightly when HIV negative cases were excluded.

Low sensitivity for syphilis POCTs has also been found by Black (2016) [34] who reported a sensitivity of 67% using the Bioline HIV/Syphilis Duo Test syphilis component, and Hess (2014) [30] reporting 47.4% sensitivity with the Chembio DPP duo test. Interestingly, Black noted that patients with a RPR titre of > 1:4 were more likely to test positive for syphilis using the Bioline POCT; in other words, when considering only those patients with a higher possibility of active syphilis as indicated by higher RPR titres, the sensitivity of Bioline POCT increased to 85%. Zorzi et al., (2017) using Bioline and Chembio single syphilis POCT, demonstrated that the higher the TPPA titre, the better the performance of the POCT’s treponemal component [16]. Our findings support these previous results in that, considering the titration provided by the laboratory tests, for TPPA titres > 1:1280 the misclassification rate for the two POCTs was extremely low. Moreover, both in this study and in Zorzi et al. [16] it is evident that when the RPR titre is equal to or more than four, the misclassification rate is also very low (our findings for RPR ≥ 4 regardless of both TPPA titre and POCT brand, are above 90%, and for RPR ≥ 8 above 94%). Thus, given that in general, the higher the confirmed elevated RPR titre, the higher the chance of active disease, we can conclude that these dual POCTs appear to have good ability in detecting probable active syphilis, i.e. both Bioline and Chembio POCTs can detect greater than 90% of probable active syphilis cases, as defined by reactive RPR and treponemal test results. Importantly, this means that there is the potential to promptly interrupt the chain of transmission amongst MSM communities. Although it is worth noting that decisions regarding the use of a threshold RPR titre (e.g. ≥ 8) should be made cautiously owing to possible recent exposure to infection and therefore failure to detect very early disease. Interpretation of all serological results should take into account patient symptoms and signs, sexual exposure and history of previous infection and treatment [16].

Given the above, it is perhaps likely that the low number of RPR positives in the UK compared to other countries could partly explain the low sensitivities for this site. For instance, of treponemal reference positive cases, 31.18% (*n* = 53/170) were RPR positive in the two Italian study sites, 63.41% (*n* = 78/123) in the Maltese site, 64.18% (*n* = 310/483) in the three Peruvian sites, but only 11.29% (*n* = 14/124) in the UK site. Clearly, many factors could play a role in the relatively poor performance of the syphilis component of both POCTs in the UK site which may require further investigation. To examine any of these issues in depth is beyond the scope of this paper but do nevertheless provide potentially important future avenues of research in relation to the performance of dual POCTs for HIV and syphilis.

Despite some site-based variations in the performance of the two index tests (sensitivity and specificity), in our study the PPV and NPV for both the HIV and syphilis components seem to be acceptable within the range of prevalence scenarios. With the exception of the syphilis component for the UK, this means that both dual POCTs could be considered as potential alternatives to standard methods of screening not only in clinics, potentially reducing waiting time and loss-to-follow-up with patients not waiting for results, but also outside of clinical settings where venepuncture may not be safe and/or laboratory testing may be challenging, such as in many resource-limited settings. Indeed, the clarity of operational instructions provided by the manufacturers for both POCTs was well understood and 100% of HCPs reported the ease of interpretation of test results as ‘fairly easy, ‘very easy’ or’unambiguous’. Thus, both Bioline HIV/Syphilis (which is on the WHO pre-qualified list of in-vitro diagnostic products), and Chembio (approved by the United States Food and Drug Administration Authority), could be extremely useful in identifying HIV and syphilis cases, in particular probable active syphilis, requiring medical assistance and treatment. Indeed, in the clinical utility arm of the broader ProSPeRo study which assessed specifically the feasibility and acceptability of the same POCTs used in this current study amongst MSM in non-clinical settings in four countries within the WHO European region, the authors found high acceptability and usability both for users and providers. The authors conclude that the implementation of dual POCTs for HIV and syphilis in non-clinical settings (namely community-based voluntary, counselling, and testing [CBVCT] services), provides an opportunity for scaling up integrated syphilis/HIV testing for MSM [40].

The strengths of our study include the large sample size of MSM participants including a sizeable proportion of positive cases, as well as generating POCT evaluation data from multiple centres and settings using standardised WHO core protocols. Our study also has some limitations. First, we enrolled a very high proportion of previously tested patients for both HIV and syphilis infection and thus our data says little about novice testers. Second, the performance results of the two POCTs must be interpreted in the light of the fact that the predictive value of a test depends on the prevalence of a particular infection and thus, the index tests may demonstrate different performance elsewhere in regions, outside of Italy, Malta, Peru, and the UK, with different prevalences in the population.

## Conclusion

The two dual POCTs evaluated in the present study, Bioline HIV/Syphilis Duo (Abbott) and DPP® HIV-Syphilis assay (Chembio), showed acceptable performance characteristics regarding sensitivity and specificity in simultaneous testing for the detection of HIV-1/2 and treponemal antibodies, amongst MSM, using a single finger prick whole blood specimen. Given the reported ease and simplicity of procedures and interpretation of test results, these dual POCTs could serve as strategic alternatives to the more demanding, and expensive conventional screening methods or single POCTs for HIV and syphilis, especially in resource-limited settings. Use of these tests in clinical and other field settings would not only simplify HIV and syphilis testing procedures, but also potentially be more cost-effective and user- friendly owing to the sole requirement for a single sample of finger-prick blood.

## Data Availability

This study which forms part of a larger programme of research co-ordinated by WHO and for which WHO acts as the repository of the ensemble of the results obtained from the individual projects. In view of this, all rights to the results of the study, including but not limited to copyright and the right to apply for, hold and exercise patent rights in respect of any invention resulting from the study, are the subject of co-ownership and responsibility between the WHO and respective country sites. Dr Igor Toskin is the Chief Investigator and contact for data availability queries (toskini@who.int).

## Abbreviations

AIDS: Acquired Immune Deficiency Syndrome
CBVCT: Community-Based Voluntary, Counselling, and Testing
CDC: Centers for Disease Control and Prevention
CLIA: Chemiluminescence Immunoassay
DOR: Diagnostic Odds Ratio
DPP: Dual Path Platform
DTS: Dried Tube Specimens
EIA: Enzyme Immunoassay
ELISA: Enzyme-Linked Immunosorbent Assay
EQA: External Quality Assessment
HCP: Health Care Provider
HIV: Human Immunodeficiency Virus
LR: Likelihood ratio
MR: Micro Reader
MSM: Men who have Sex with Men
NPV: Negative Predictive Values
POCT: Point-of-Care Test
PPV: Positive Predictive Values
PrEP: Pre-Exposure Prophylaxis
ProSPeRo: Project on Sexually Transmitted Infection Point-of-Care Testing
QUADAS: Quality Assessment of Diagnostic Accuracy Studies
RPR: Rapid Plasma Reagin
STARD: Standards for Reporting Studies of Diagnostic Accuracy
STIs: Sexually Transmitted Infections
TPPA: *Treponema pallidum* Passive Particle Agglutination
TPHA: *Treponema pallidum* Hemagglutination Assay
WB: Western Blot
WHO: World Health Organization
